# Elevated In Vivo Levels of a Single Transcription Factor Directly Convert Satellite Glia into Oligodendrocyte-like Cells

**DOI:** 10.1371/journal.pgen.1005008

**Published:** 2015-02-13

**Authors:** Matthias Weider, Amélie Wegener, Christian Schmitt, Melanie Küspert, Simone Hillgärtner, Michael R. Bösl, Irm Hermans-Borgmeyer, Brahim Nait-Oumesmar, Michael Wegner

**Affiliations:** 1 Institut für Biochemie, Emil-Fischer-Zentrum, Friedrich-Alexander-Universität Erlangen-Nürnberg, Erlangen, Germany; 2 Experimentelle Biomedizin, Rudolf-Virchow-Zentrum, Universitätsklinikum Würzburg, Würzburg, Germany; 3 ZMNH, Universitätsklinikum Eppendorf, Hamburg, Germany; 4 Institut du Cerveau et de la Moelle Epinière, ICM, Inserm U1127, Université Pierre et Marie Curie, Sorbonne Paris Cité, UMR-S1127, CNRS UMR 7225, Paris, France; Stanford University School of Medicine, UNITED STATES

## Abstract

Oligodendrocytes are the myelinating glia of the central nervous system and ensure rapid saltatory conduction. Shortage or loss of these cells leads to severe malfunctions as observed in human leukodystrophies and multiple sclerosis, and their replenishment by reprogramming or cell conversion strategies is an important research aim. Using a transgenic approach we increased levels of the transcription factor Sox10 throughout the mouse embryo and thereby prompted Fabp7-positive glial cells in dorsal root ganglia of the peripheral nervous system to convert into cells with oligodendrocyte characteristics including myelin gene expression. These rarely studied and poorly characterized satellite glia did not go through a classic oligodendrocyte precursor cell stage. Instead, Sox10 directly induced key elements of the regulatory network of differentiating oligodendrocytes, including Olig2, Olig1, Nkx2.2 and Myrf. An upstream enhancer mediated the direct induction of the *Olig2* gene. Unlike Sox10, Olig2 was not capable of generating oligodendrocyte-like cells in dorsal root ganglia. Our findings provide proof-of-concept that Sox10 can convert conducive cells into oligodendrocyte-like cells in vivo and delineates options for future therapeutic strategies.

## Author Summary

Developmental or acquired defects of oligodendrocytes or their myelin sheaths impairs saltatory nerve conduction in the central nervous system and thus leads to severe neurological diseases. Strategies to regenerate or replace these cells require a deeper understanding of the regulatory processes that underlie their generation during development. Here we show in a Sox10 overexpressing mouse model that increase of the levels of a single transcription factor during embryogenesis efficiently converts the already Sox10 expressing satellite glial cells of the peripheral nervous system into oligodendrocyte-like cells by a mechanism that does not simply recapitulate developmental oligodendrogenesis but involves direct Sox10-dependent induction of the oligodendroglial differentiation network. Our study identifies mechanisms that may help to convert other cell types into oligodendrocytes and thus prove eventually useful for therapies of myelin diseases.

## Introduction

Transcription factor-mediated reprogramming is currently the method of choice for the generation of induced pluripotent stem (iPS) cells [1]. It is also used to directly convert one cell type into another. Successful conversion depends on the choice of transcription factors, but is also influenced by the proteomic constitution of the targeted cell with some cells being more susceptible to acquiring a specific new identity than others [2]. Both reprogramming and conversion are usually performed in culture with low efficiencies and are rarely studied in vivo.

Recently, murine fibroblasts have been converted into oligodendrocyte precursor cells (OPC) which in turn had the capacity to differentiate into myelinating oligodendrocytes when transplanted into the brain of a myelin-deficient mouse mutant [3,4]. This feat is important as generation of oligodendroglial cells from iPS cells is relatively inefficient and time-consuming [5]. Once optimized and adopted to human cells, it offers a potential source for cell replacement strategies in the various demyelinating and dysmyelinating diseases.

The conversion to OPC was achieved by applying a cocktail of several transcription factors to fibroblasts. While one study settled on a set of eight transcription factors with the core group consisting of Sox10, Olig2 and Nkx6.2 [3], the other defined a three-factor mix of Sox10, Olig2 and Zfp536 [4]. Sox10 and Olig2 thus seem to represent the minimal common denominator for the conversion process. The key role of Sox10 and Olig2 is not unexpected as previous studies had shown the exceptional importance of both transcription factors for oligodendroglial development and myelin formation during embryonic and postnatal development [6,7,8,9,10]. Olig2 is largely restricted to oligodendroglial cells. The few other Olig2-expressing cell populations (i.e. neuroepithelial cells of the ventral ventricular zone, motoneuron precursors and a subset of astrocyte precursors) are transient and restricted to the embryonic and early postnatal central nervous system (CNS) [11]. Sox10, in contrast, additionally occurs in several other cell types outside the CNS which are mostly neural crest-derived, such as all glial cells of the peripheral nervous system (PNS) [12].

When tested as single factors for their ability to induce OPC features in fibroblasts, only Sox10, but not Olig2 was found to exhibit some activity [4]. An independent study on cultured human neural progenitor cells recently confirmed Sox10 as the principle and rate-limiting determinant of myelinogenic fate [13]. This prompted us to postulate that it might be possible to induce oligodendrocyte properties in vivo in an especially conducive cell type with Sox10 alone. Indeed we found that its overexpression in already Sox10-positive satellite glia of PNS dorsal root ganglia (DRG) is sufficient to generate oligodendrocyte-like cells in vivo. The available evidence indicates that a key element in this conversion process is the activation of Olig2 as the second essential oligodendroglial identity factor mediated by a Sox10-responsive evolutionarily conserved enhancer of the Olig2 gene. Interestingly, analogous overexpression of Olig2 is not sufficient to convert satellite glia into oligodendrocyte-like cells. Our findings provide proof-of-concept that Sox10 can be used to convert a conducive cell type into oligodendrocyte-like cells in vivo and delineates options for future therapeutic strategies.

## Results

### Overexpression of Sox10 in dorsal root ganglia leads to the appearance of oligodendrocyte-like cells

**Fig 1:**
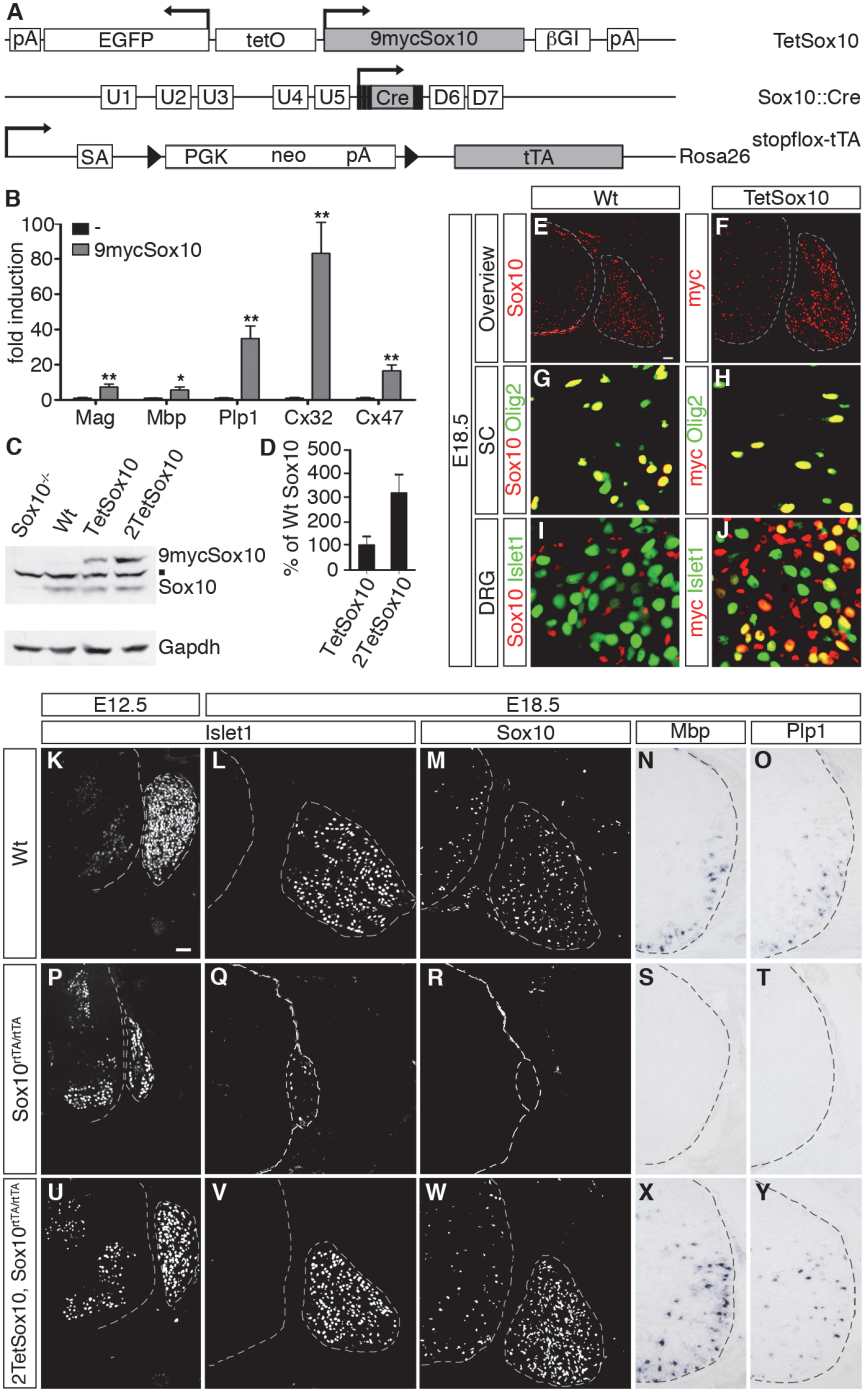
Transgenic Sox10 is overexpressed in normally Sox10 expressing cells and functional. (A) Schematically shown are the *TetSox10*, *Sox10*::*Cre* and *Rosa26* ^*stopflox-tTA*^ alleles that in combination allow Sox10 overexpression at its sites of natural occurrence. Arrows mark transcription start sites, triangles loxP sites. 9mycSox10, coding sequences for myc-tagged Sox10; βGI, β-globin intron; Cre, Cre recombinase coding sequences; D6 and D7, downstream *Sox10* enhancers; EGFP, coding sequences for enhanced green fluorescent protein; neo, neomycin resistance cassette; pA, polyadenylation site; PGK, phosphoglycerate kinase gene promoter; SA, splice acceptor; tetO, bidirectional tetracycline-responsive promoter; tTA, coding sequences for tetracycline-controlled transactivator; U1-U5, upstream *Sox10* enhancers. **(B)** To test functionality of myc-tagged Sox10 in vitro, transient transfection were carried out in Neuro2a cells with reporter plasmids that carried the luciferase gene under control of known Sox10-responsive regulatory elements in the absence or presence of 9mycSox10 expression plasmid. Sox10-responsive regulatory elements included the promoters of the *Mag*, *Mbp*, *Cx32* and *Cx47* genes as well as the intronic *Plp1* enhancer. Luciferase activities were determined in three experiments each performed in duplicates. The activity obtained for the luciferase reporter in the absence of ectopic transcription factor was arbitrarily set to 1. Fold inductions for Sox10 were calculated in relation and are presented as mean ± SEM. Reporter activation by myc-tagged Sox10 was statistically significant in all cases (Student’s t-test; *, P ≤0.05; **, P ≤0.01). **(C, D)** Amounts of wildtype (Sox10) and myc-tagged (9mycSox10) protein were analyzed by Western blot in brain extracts of wildtype (Wt), Sox10-deficient (Sox10^-/-^), TetSox10 and 2TetSox10 embryos at E18.5. A representative Western blot is shown in C including the Gapdh normalization control, while D represents the quantification from three independent biological replicates (Student’s t-test; P ≤0.05) with the amounts of wildtype Sox10 set to 100% and the amounts of myc-tagged Sox10 expressed in relation to this (± SEM). The square in C marks a cross-reactive band. **(E-J)** To compare the expression pattern of wildtype and myc-tagged Sox10 at E18.5, IHC was performed with antibodies directed against Sox10 (red) on transverse sections of wildtype (Wt) embryos (E, G, I) and anti-myc-tag antibodies (red) on corresponding sections of TetSox10 embryos (F, H, J), both times in combination with antibodies against the oligodendroglial marker Olig2 and the neuronal marker Islet1 (both in green). In addition to an overview (E, F), magnifications are shown for spinal cord (SC in G, H) and DRG (I, J). **(K-Y)** To test functionality of myc-tagged Sox10 in vivo, an attempt was made to rescue PNS and oligodendrocyte differentiation defects in Sox10-deficient mice (Sox10^rtTA/rtTA^) by simultaneous overexpression of myc-tagged Sox10 (2TetSox10, Sox10^rtTA/rtTA^). Wildtype (Wt, in K-O) and Sox10^rtTA/rtTA^ (P-T) mice served as controls for 2TetSox10, Sox10^rtTA/rtTA^ (U-Y) mice. Phenotypic analysis was by IHC and ISH on transverse sections (thoracic level) at E12.5 (K, P, U) and E18.5 (L-O, Q-T, V-Y) using antibodies directed against Islet1 (K, L, P, Q, U, V) and Sox10 (M, R, W) as well as riboprobes for *Mbp* (N, S, X) and *Plp1* (O, T, Y). Size bars: 50 μm in E (valid for E-J) and K (valid for K-Y).

For targeted and strictly controlled Sox10 expression in vivo a transgene was generated in which rat Sox10 cDNA was placed under control of a bidirectional tetracycline-responsive promoter (Fig. 1A). GFP expression from the same promoter and Sox10-tagging with an aminoterminal 9myc epitope were used for detection of transgene expression. Luciferase reporter gene assays in transiently transfected Neuro2A cells confirmed that the aminoterminal 9myc tag did not interfere with the ability of Sox10 to activate a series of its targets, including the promoters of the *Mag* (myelin associated glycoprotein), *Mbp* (myelin basic protein), *Cx32* (connexin 32), *Cx47* (connexin 47) genes and the intronic oligodendrocyte enhancer of the *Plp1* (proteolipid protein 1) gene (Fig. 1B).

Of the founders obtained by pronucleus injection of this *TetSox10* transgene one was expanded into a line. It contained less than 5 tandem copies of the transgene on the long arm of mouse chromosome 10 (10qD1). In this study it was mostly combined with a *Rosa26* ^*stopflox-tTA*^ [14] and a *Sox10*::*Cre* [15] allele to direct transgene expression to all cells that normally express Sox10 during development or in the adult (Fig. 1A). Brain extracts from *Rosa26* ^*+/stopflox-tTA*^ *Sox10*::*Cre* mice contained as much transgenic as wildtype Sox10 at embryonic day (E) 18.5 when they were hemizygous for TetSox10, and approximately three times as much when homozygous (i.e. 2TetSox10) (Fig. 1C, D). Transgenic Sox10 expression corresponded to sites of endogenous Sox10 expression (Fig. 1E, F). In spinal cord and other CNS areas, both endogenous and transgenic Sox10 were restricted to and present in the vast majority of Olig2-positive cells of the oligodendroglial lineage (Fig. 1G, H). In the PNS, both DRG and nerves were labelled similarly by an anti-Sox10 antibody in the wildtype and an anti-myc-tag antibody in the transgenic animal (Fig. 1E, F, I, J). However, while endogenous Sox10 was restricted to glial cells as previously shown [16], transgenic Sox10 was additionally found in a subset of DRG neurons as a relic of their ontogenetic history (Fig. 1I, J). DRG neurons stem from Sox10-positive neural crest precursor cells and therefore experience transient *Sox10*::*Cre* expression which triggers induction of the *TetSox10* transgene. The continued presence of transgenic Sox10 in cell lineages that normally express the protein only transiently and the resulting developmental defects may be one reason for the very early postnatal death of *Sox10*::*Cre* induced, tTA expressing TetSox10 and 2TetSox10 mice.

It had previously been shown that *Sox10* deletion leads to cell loss and disorganization within the PNS, including a dramatic reduction of DRG size [16]. The CNS is less affected and mainly suffers from absent oligodendroglial differentiation and myelination [8,9]. When transgenic Sox10 is expressed homozygously on an otherwise Sox10-deficient background (i.e. in *2TetSox10* under control of *Sox10* ^*rtTA*^ in *Sox10* ^*rtTA/rtTA*^ mice [17] following doxycycline treatment), these defects are rescued as indicated by a near normal DRG size and reappearance of myelin gene expression in the spinal cord of compound mutant embryos (compare Fig. 1K-O to Fig. 1P-T and Fig. 1U-Y). This confirms functionality of the 9myc-tagged transgenic Sox10 in vivo.

**Fig 2:**
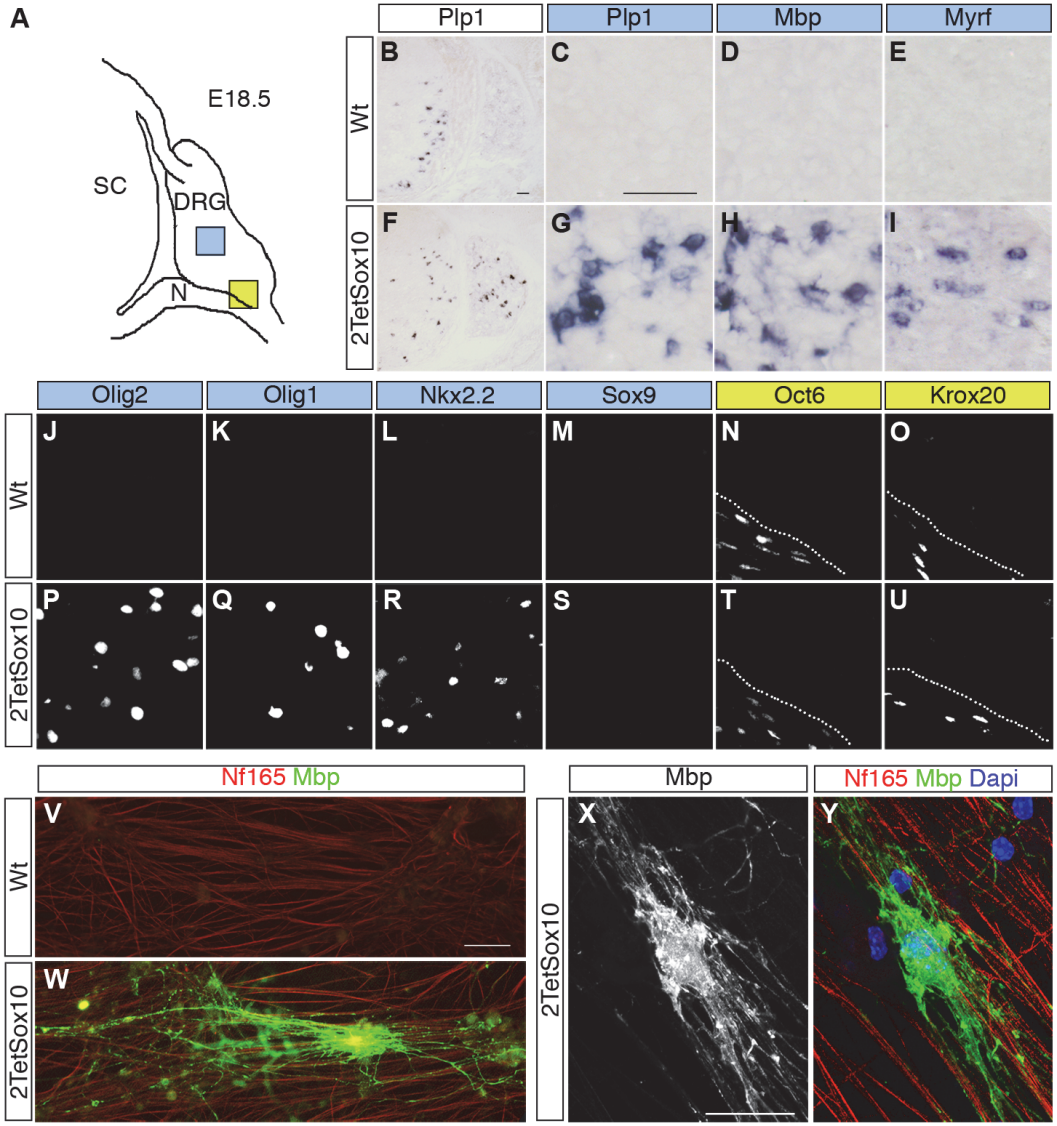
Sox10 overexpression in DRG leads to appearance of oligodendrocyte-like cells. **(A)** Spinal cord (SC), DRG and peripheral nerves (N) are schematically shown at E18.5. Areas shown in C-E and G-U are marked as blue and yellow squares. **(B-U)** ISH and IHC were carried out on transverse sections (thoracic level) of wildtype (Wt) and 2TetSox10 embryos at E18.5 with riboprobes against *Plp1* (B, C, F, G), *Mbp* (D, H) and *Myrf* (E, I) or antibodies directed against Olig2 (J, P), Olig1 (K, Q), Nkx2.2 (L, R), Sox9 (M, S), Oct6 (N, T) and Krox20 (O, U). The areas from which pictures were taken are indicated by the blue or yellow color in the box above the panels. Color coding is according to the scheme in A. Stippled lines in N, O, T, U correspond to the nerve-DRG boundary. **(V-Y)** Dissociated cells from DRG of E14.5-old wildtype (V) and 2TetSox10 (W-Y) mouse embryos were co-cultured with rat DRG neurons for 4 weeks under myelinating conditions before immunocytochemical staining with Nf165 (red) and Mbp (green in V, W, Y and white in X). Nuclei were counterstained in Y with 4,6-diamidino-2-phenylindole (Dapi) (blue). W, X, Y show an example of a myelinating oligodendrocyte photographed with a Leica DMI6000 B inverted microscope equipped with a DFC350 FX camera (V, W) or a Zeiss LSM 780 confocal microscope (X, Y). Size bars: 50 μm in B (valid for B, F), C (valid for C-E, G-U) and V (valid for V, W); 20 μm in X (valid for X, Y).

When analysing oligodendroglial development in late embryos that overexpress *TetSox10* and *2TetSox10* under control of *Sox10*::*Cre* and *Rosa26* ^*stopflox-tTA*^ we observed an earlier appearance of myelin markers such as *Plp1* and *Mbp* in spinal cord and other CNS regions. This may be indicative of a precocious oligodendrocyte differentiation. More intriguingly, *Plp1* and *Mbp* expressing cells were also detected in substantial numbers in DRG of 2TetSox10 embryos at E18.5, whereas they were rare in DRG of TetSox10 mice and absent from age-matched wildtype embryos (Fig. 2B-D, F-H). Myelin gene expression in DRG of 2TetSox10 embryos went along with the selective presence of Myrf, Olig2, Olig1 and Nkx2.2 (Fig. 2E, I-L, P-R). These transcription factors are strongly associated with oligodendrocytes and oligodendroglial myelination, while absent from Schwann cells, the only cell type normally capable of myelination in the PNS. In contrast, markers of myelinating Schwann cells such as Oct6 and Krox20 transcription factors were not expressed in DRG and remained restricted to the peripheral nerves of 2TetSox10 embryos (Fig. 2N, O, T, U).

Dissociated cells from DRG of E14.5 2TetSox10 mouse embryos also gave rise to Mbp-positive cells with the typical morphology of myelinating oligodendrocytes at low frequency when co-cultured with rat DRG neurons under myelinating conditions (Fig. 2W-Y). Such cells were not observed when dissociated DRG of E14.5 wildtype embryos were used instead (Fig. 2V). We therefore conclude that the myelinating cells in DRG of 2TetSox10 mice closely resemble oligodendrocytes. As we cannot exclude the possibility that these cells retain differences to oligodendrocytes and as it is technically not feasible for us to collect enough of these cells for in-depth expression profiling and characterization, we will refer to them as oligodendrocyte-like cells.

### Satellite glia are the source of oligodendrocyte-like cells

Despite the strong expression of oligodendrocyte lineage and differentiation markers, oligodendrocyte precursor cell (OPC) markers such as Sox9, Pdgfra and NG2 were not detected in substantial levels in DRG of 2TetSox10 mice (e.g. Fig. 2M, S). This leads to the conclusion that these oligodendrocyte-like cells have not gone through a classical OPC stage and may be derived from another cell source.

**Fig 3:**
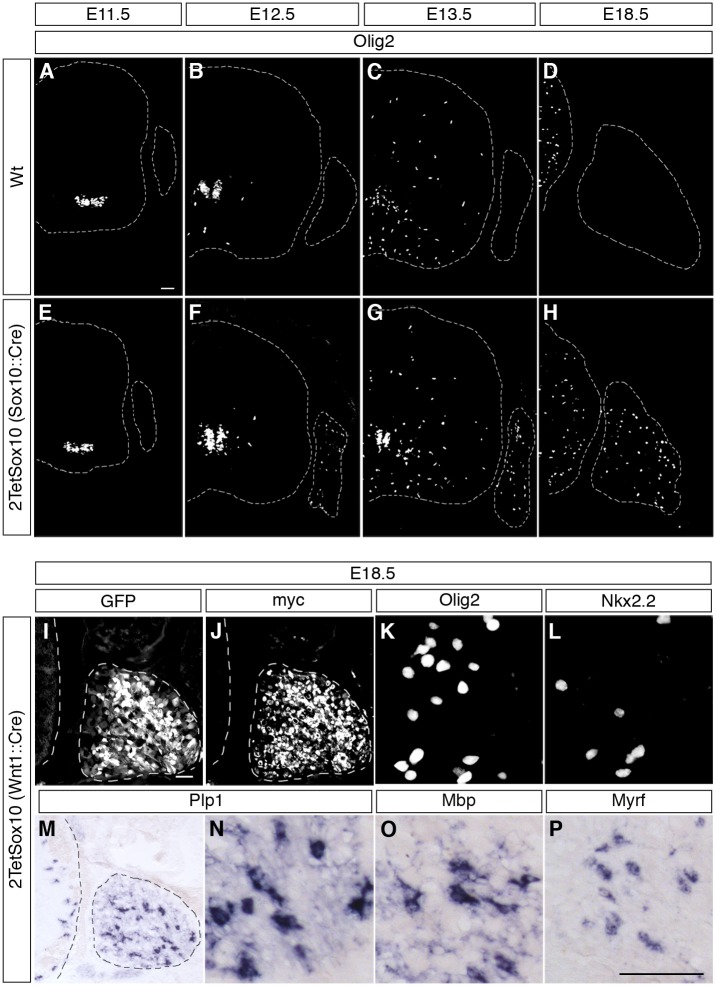
Oligodendrocyte-like cells arise from neural crest-derived cells within the DRG. **(A-H)** To follow the appearance of oliogdendroglial cells during embryonic development IHC was carried out on transverse sections (thoracic level) of wildtype (Wt) and 2TetSox10 embryos at E11.5 (A, E), E12.5 (B, F), E13.5 (C, G) and E18.5 (D, H) with antibodies against Olig2. Contours of spinal cord and DRG are marked by stippled lines. **(I-P)** IHC and ISH were also performed at E18.5 on embryos in which two copies of the *TetSox10* transgene were activated by *Wnt1*::*Cre* instead of *Sox10*::*Cre* using antibodies directed against GFP (I), myc-tag (J), Olig2 (K), and Nkx2.2 (L), or riboprobes directed against *Plp1* (M, N), *Mbp* (O) and Myrf (P). Panels K, L, N-P show regions within the DRG. Size bars: 50 μm in A (valid for A-H), I (valid for I, J, M) and P (valid for K, L, N-P).

A study of consecutive embryonic stages from E11.5 to E18.5 (Fig. 3A-H) revealed that ectopic Olig2 expressing cells in DRG of 2TetSox10 mice are not yet detectable at E11.5, but are already present at E12.5 (Fig. 3E, F) approximately the same time when OPC start to be generated in the ventral ventricular zone and emigrate from the pMN domain into the marginal zone of the spinal cord (Fig. 3A, B, E, F). This early appearance strongly argues for an origin of Olig2-positive cells in DRG of 2TetSox10 mice that is independent from OPC and outside the CNS.

Considering that most of the PNS is neural crest-derived and that *Wnt1*::*Cre* is widely active throughout the early neural crest, we exchanged *Sox10*::*Cre* for this Cre driver to induce 2TetSox10 expression. Analysis of GFP autofluorescence as well as direct detection of transgenic Sox10 by anti-myc antibodies confirmed the widespread activation and expression of the transgenic construct throughout the embryonic PNS (Fig. 3I, J). It went along with efficient generation of differentiating oligodendrocyte-like cells in DRG as evident from the induced expression of Olig2, Nkx2.2, Myrf, Plp1 and Mbp (Fig. 3K-P). We therefore conclude that the oligodendrocyte-like cells stem from neural crest-derived cells of the PNS.

**Fig 4:**
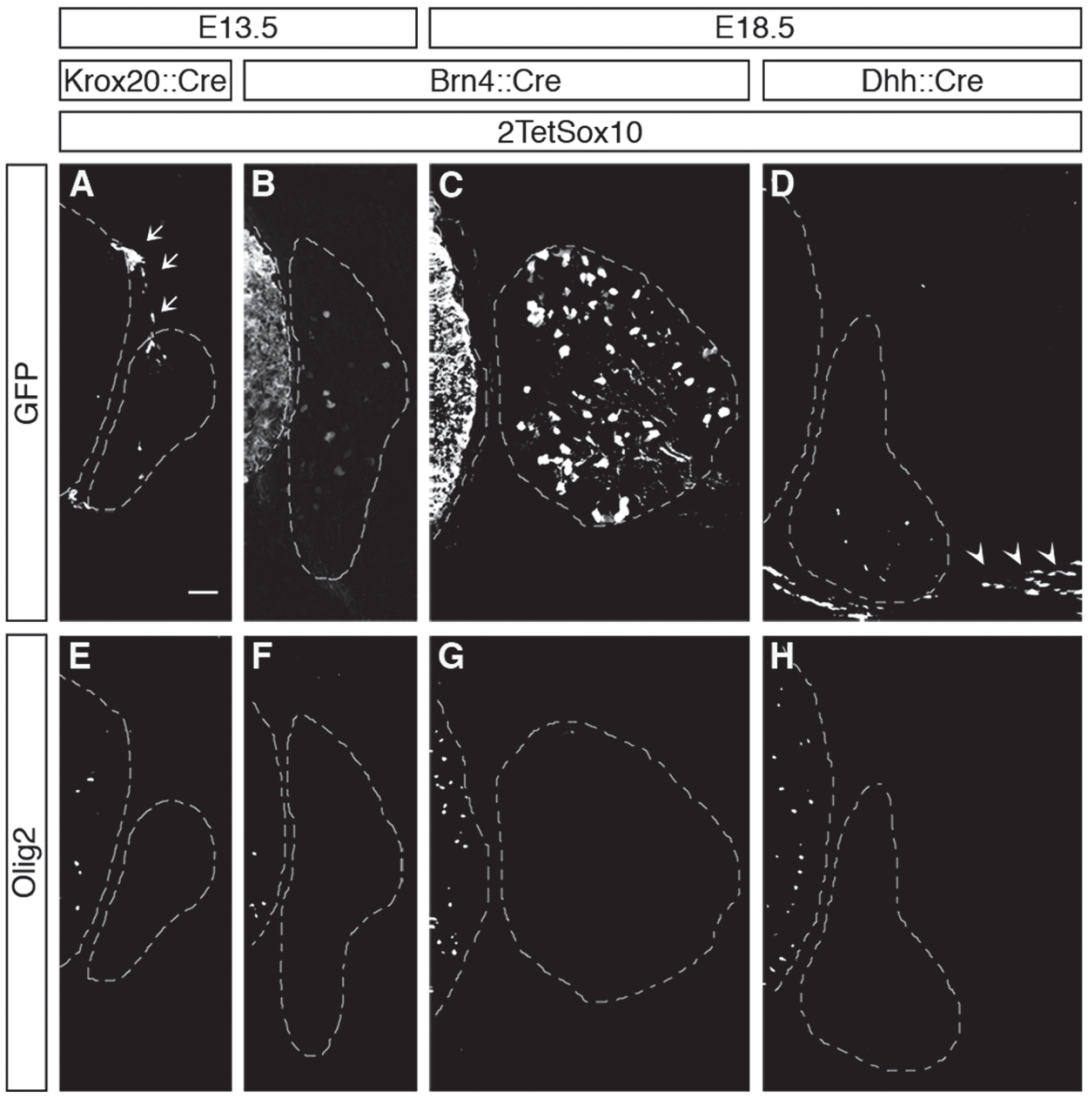
Oligodendrocyte-like cells in DRG do not arise from boundary cap cells, Schwann cells or peripheral neurons. IHC was carried out with antibodies against GFP (A-D) or Olig2 (E-H) at E13.5 and E18.5 on transverse sections of embryos in which two copies of the *TetSox10* transgene were activated by *Krox20*::*Cre* (A, E), *Brn4*::*Cre* (B, C, F, G), or *Dhh*::*Cre* (D, H) instead of *Sox10*::*Cre*. Contours of spinal cord and DRG are marked by stippled lines. Boundary cap cells are marked by arrows in A, Schwann cells by arrowheads in D. Size bar in A, valid for A-H: 50 μm.

**Fig 5:**
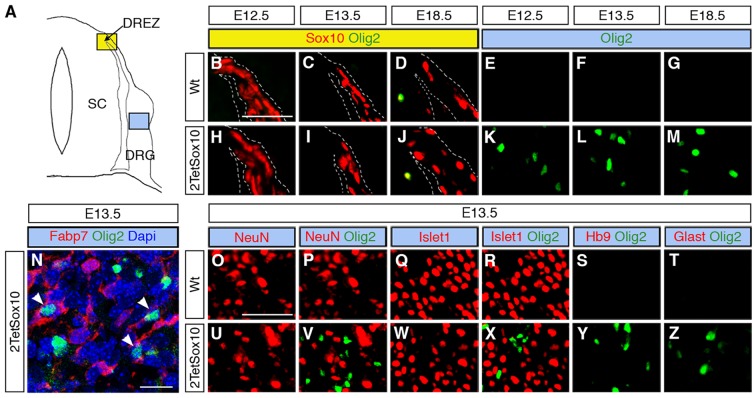
Ectopic Olig2-positive cells in the DRG express peripheral glial, but not neuronal or astrocytic marker and are not derived from boundary cap cells. **(A)** Spinal cord (SC), DRG, dorsal root entry zone (DREZ) and peripheral nerves are schematically depicted. Areas shown in B-Y are marked as blue and yellow squares. **(B-Z)** Co-IHC was carried out on transverse sections (thoracic level) of wildtype (Wt, B-G and O-T) and 2TetSox10 (H-N and U-Z) embryos at E12.5, E13.5 and E18.5 with antibodies directed against Olig2 (B-N, P, R-T, V, X-Z, green), Sox10 (B-D, H-J, red), Fabp7 (N), NeuN (O, P, U, V), Islet1 (Q, R, W, X), Hb9 (S, Y) and Glast (T, Z). The areas from which pictures were taken are indicated by the blue or yellow color in the box above the panels. Color coding is according to the scheme in A. DREZ and spinal cord perimeter are demarcated by stippled lines in B-D and H-J. In N, nuclei were counter-stained with Dapi. Panels B-M and O-Z were photographed with a Leica DMI6000 B inverted microscope equipped with a Leica DFC350 FX camera, whereas panel N was taken with a Zeiss LSM 780 confocal microscope. Size bar: 50 μm in B and O (valid for B-M and O-Z, respectively); 20 μm in N.

Because boundary cap cells represent a versatile source for different neural crest-derived cell types in the PNS [18] and have been reported to give rise to oligodendrocytes after engraftment into the CNS [19], we checked whether these cells were the source of Olig2-positive cells in the DRG. A *Krox20*::*Cre* driver in combination with *Rosa26* ^*stopflox-tTA*^ allows a restricted induction of 2TetSox10 in this transient cell population which is localized at the dorsal root entry zone during early embryonic times (Fig. 4A, see arrows). However, such selective induction of transgenic Sox10 expression did not lead to the appearance of Olig2-positive cells in DRG (Fig. 4E). Furthermore, Olig2-positive cells were never observed in substantial numbers in the dorsal root entry zone of mice in which 2TetSox10 expression was under control of *Sox10*::*Cre* at times when they were already numerous in the DRG (compare Fig. 5A-G to Fig. 5H-M). This argues against a boundary cap derived-origin of the ectopic Olig2-positive cells.

*Brn4*::*Cre* is active throughout the early CNS and in DRG neurons [20,21]. When this Cre line was used to activate 2TetSox10 expression (Fig. 4B, C) we again failed to observe any Olig2-positive cells in the DRG (Fig. 4F, G). This finding not only provides additional evidence for an origin of the Olig2-positive cells outside the CNS, it also excludes DRG neurons as source. The latter finding is also supported by the fact that there was no co-labelling of Olig2-positive cells with NeuN or Islet1 as markers for PNS neurons in DRG of mice in which 2TetSox10 expression was under control of *Sox10*::*Cre* (Fig. 5O-R, U-X). Instead, we observed a substantial overlap of Olig2 and Fabp7 staining in DRG of 2TetSox10 mice at E13.5 (Fig. 5N). Considering that Fabp7 is the only reliable early marker for peripheral glia, we conclude that Olig2 induction occurs in glial cells of the DRG. Interestingly, Fabp7 co-staining was only observed in cells with weak, but not with strong Olig2 immunoreactivity arguing that co-expression is transient and restricted to the phase of Olig2 induction.

Finally, we employed *Dhh*::*Cre* in combination with *Rosa26* ^*stopflox-tTA*^ to induce 2TetSox10 expression. *Dhh*::*Cre* is active in the Schwann cell lineage from the precursor stage onwards. Although we efficiently activated transgene expression in Schwann cells, for instance in spinal nerves in the immediate vicinity of the DRG (Fig. 4D, see arrowheads), no Olig2-positive cells were generated in the DRG itself (Fig. 4H). Considering (i) that the Olig2-positive cells are derived from PNS cells other than boundary cap cells, Schwann cells or DRG neurons and (ii) that they are glial in origin, satellite glia within the DRG remain as sole source. We thus conclude that overexpression of Sox10 in satellite glia leads to the generation of differentiating and myelinating oligodendrocyte-like cells. This conversion seems specific as we failed to obtain any evidence for a simultaneous generation of astrocytes or spinal cord neurons in DRG upon Sox10 overexpression in 2TetSox10 mice (Fig. 5, S, T, Y, Z)

### Sox10 directly activates Olig2 in satellite glia as a key event in their conversion to oligodendrocyte-like cells

**Fig 6:**
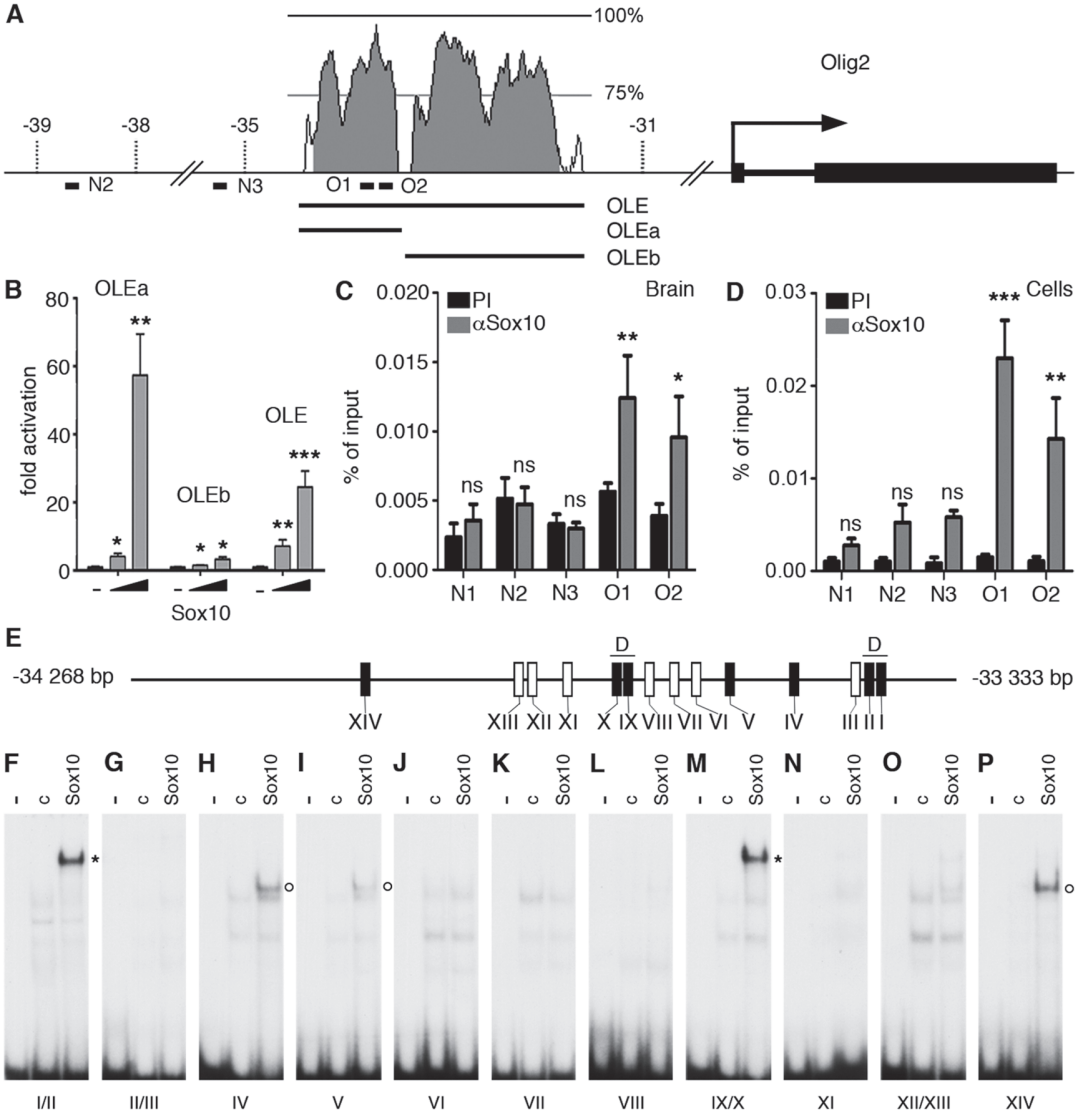
The evolutionary conserved OLE and its OLEa subfragment are directly activated and bound by Sox10. **(A)** Shown are position and conservation profile (from 50% to 100% between mouse and human) of the evolutionarily conserved OLE enhancer within the mouse *Olig2* genomic locus. *Olig2* exons are shown as black boxes. Numbers refer to the distance in kbp relative to the transcriptional start site (marked by arrow). OLE subfragments OLEa and OLEb and the position of PCR fragments N2, N3, O1, O2 for ChIP studies are highlighted. **(B)** Sox10-dependent activation of OLE and its subfragments was studied in Neuro2a cells by cotransfection of increasing amounts of Sox10 expression plasmids (50 ng and 500 ng per 3.5 cm plate) with reporter plasmids that carried the luciferase gene under control of a minimal promoter in combination with OLE, OLEa or OLEb. Luciferase activities were determined in four experiments each performed in duplicates. The activity obtained for the luciferase reporter in the absence of ectopic transcription factor was arbitrarily set to 1. Fold inductions for Sox10 were calculated in relation and are presented as mean ± SEM. Activation of luciferase reporters by Sox10 was statistically significant in all cases (Student’s t-test; *, P ≤0.05; **, P ≤0.01; ***, P ≤0.001). **(C, D)** ChIP was performed on brain of three weeks old mice (C) and primary cultures of differentiated oligodendrocytes (D) using antibodies directed against Sox10 (αSox10) and control preimmune serum (PI). Quantitative PCR was applied on the immunoprecipitate to detect regions O1, O2, N2 and N3 from the *Olig2* genomic region as well as N1 from rat chromosome 9q38 or syntenic mouse chromosome 17qE5. Values for each fragment correspond to the percentage of material precipitated from the input and represent the mean ± SEM of four biological replicates. Statistical significance of amounts precipitated with αSox10 relative to PI was determined by two way analysis of variance (ANOVA) with Bonferroni post tests (ns, not significant; *, P ≤0.05; **, P ≤0.01; ***, P ≤0.001). **(E)** Within OLEa (positions-34268 to-33333 relative to the *Olig2* transcriptional start) 14 potential binding sites I-XIV were detected (see also Fig. 7). Of these sites only I, II, IV, V, IX, X, and XIV bound substantial amounts of Sox10 in vitro (black boxes) either as dimers (I/II and IX/X, marked by D) or monomers (IV, V, XIV). **(F-P)** EMSA was performed with radiolabelled double-stranded oligonucleotides encompassing one or two closely spaced putative Sox10 binding sites from OLEa. Oligonucleotides were incubated in the absence (-), or presence (c, Sox10) of protein extracts before gel electrophoresis as indicated above the lanes. Extracts were from mock-transfected HEK293 cells (c) or HEK293 cells expressing full length Sox10 (Sox10). Oligonucleotides with site B and site C/C‘ from the promoter of the *Mpz* (myelin protein zero) gene served as positive control for Sox10 monomer and dimer binding [48]. Positions of bands indicative of Sox10 binding are marked by asterisks for dimers and circles for monomers.

It seemed reasonable to assume that one of the earliest events during the conversion of satellite glia into oligodendrocyte-like cells should be the Sox10-dependent activation of Olig2 as an essential determinant of oligodendroglial identity. During oligodendrocyte specification in the CNS, Olig2 is genetically upstream of Sox10 and appears to be a direct activator of *Sox10* gene expression [22,23,24]. However, it has also been proposed that later on during oligodendrocyte development, Sox10 may in turn help to maintain Olig2 expression [25]. An increase of overall Sox10 levels in satellite glia upon transgene expression could thus be sufficient to activate Olig2 expression and thereby establish a key circuit of the oligodendrocyte regulatory network. To study this hypothesis, we searched for evolutionarily conserved non-coding regions (ECR) in the vicinity of the *Olig2* gene. One such ECR was recently shown to be active in the early spinal cord, but was not analyzed at times relevant for oligodendrocyte development [26]. This 2.7 kb Olig2 ECR (OLE) is localized approximately 33 kb upstream of the transcriptional start of the mouse *Olig2* gene (Fig. 6A). It furthermore exhibited a robust response to the presence of Sox10 in transiently transfected Neuro2A cells and allowed a 25-fold Sox10-dependent activation of a luciferase reporter gene (Fig. 6B). When split into a more distal (OLEa) and a more proximal part (OLEb), OLEa retained Sox10 responsiveness and even elicited an increased activation of the luciferase reporter, whereas OLEb failed to do so arguing that OLEa may contain the core elements for Sox10 induction. In agreement, chromatin immunoprecipitation (ChIP) experiments on three-week old mouse brain and oligodendrocytes differentiated in culture for 6 days found a specific enrichment of OLEa in chromatin precipitated with α-Sox10 antibodies (Fig. 6C, D) arguing that the effect of Sox10 on OLEa is direct.

**Fig 7:**
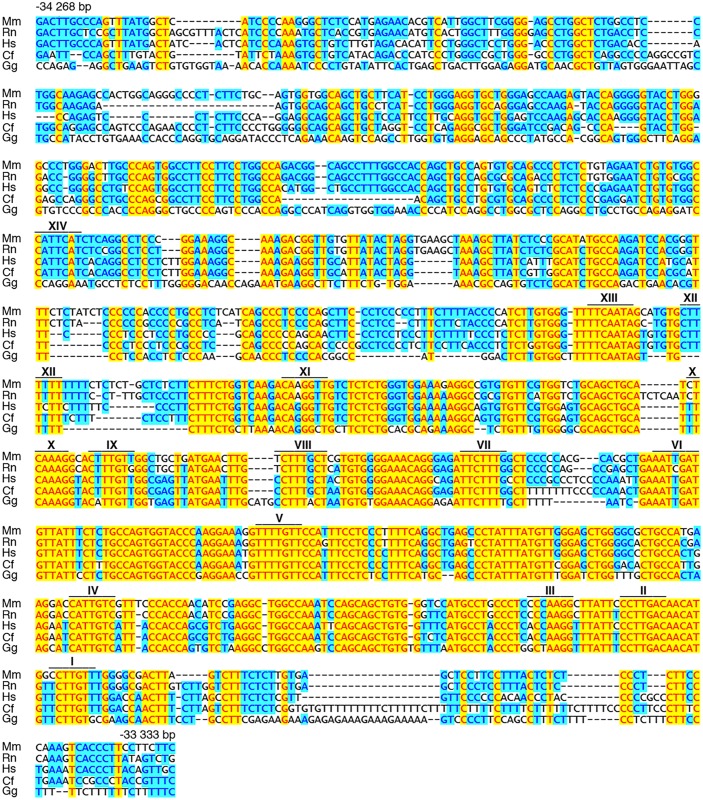
OLEa contains multiple evolutionary conserved putative Sox10 binding sites. Mouse (Mm) OLEa sequence from positions-34268 to-33333 bp relative to the *Olig2* transcriptional start are compared to OLEa sequences from rat (Rn), human (Hs), dog (Cf) and chicken (Gg). Fully conserved nucleotides are marked in yellow, mostly conserved ones in blue. Putative Sox10 binding sites are marked by a bar and a Roman numeral above the sequence.

**Fig 8:**
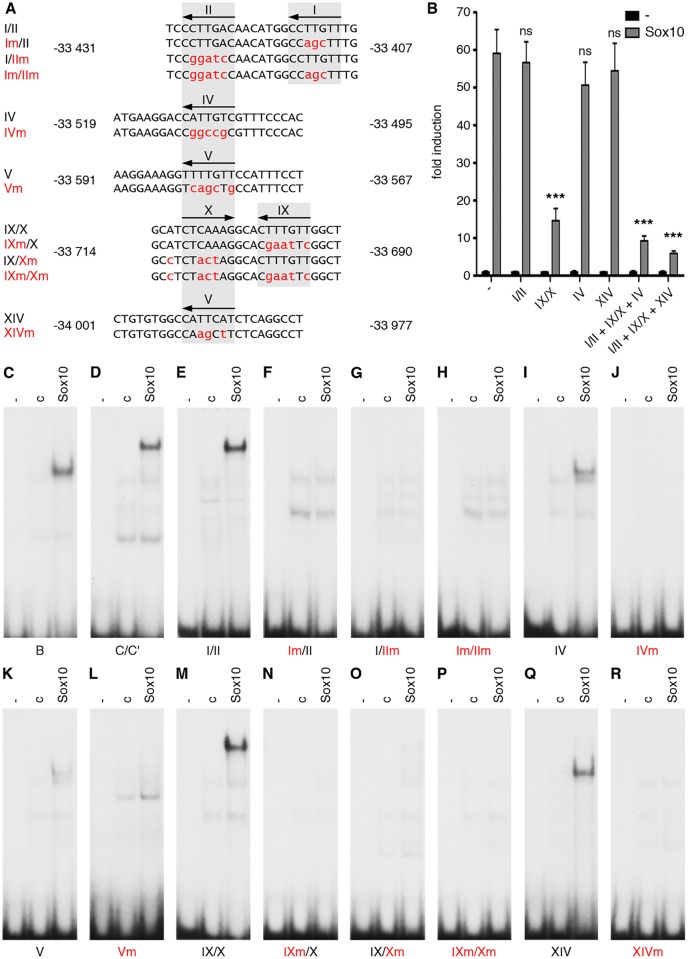
Sox binding sites mediate OLEa responsiveness to transcriptional activation by Sox10. **(A)** Three or more nucleotides (small red letters) were changed within the Sox binding sites I/II, IV, V, IX/X and XIV of OLEa to generate mutant sites. Numbers on both sides of the sequence indicate the exact position relative to the transcriptional start. Location of Sox sites are highlighted in grey and orientations indicated by arrows. **(B)** Sox binding site mutations were introduced in several combinations into the OLEa reporter plasmid and luciferase assays were performed after transient transfection of this plasmid in Neuro2A cells in the absence or presence of Sox10 expression plasmid. The Sox binding sites mutated in each reporter construct are given below the lanes. Luciferase activities were determined in four experiments each performed in duplicates. The activity obtained for each luciferase reporter in the absence of Sox10 was arbitrarily set to 1. Fold inductions for Sox10 were calculated in relation and are presented as mean ± SEM. Statistical significance of fold inductions obtained with mutant reporters relative to wildtype was determined by one way analysis of variance (ANOVA) with Tukey’s multiple comparison post test (ns, not significant; ***, P ≤0.001). **(C-R)** EMSA was performed with radiolabelled double-stranded oligonucleotides containing Sox binding sites in wildtype (same gels as in Fig. 6) or mutant version. Oligonucleotides were incubated in the absence (-), or presence (c, Sox10) of protein extracts before gel electrophoresis as indicated above the lanes. Extracts were from mock-transfected HEK293 cells (c) or HEK293 cells expressing full length Sox10 (Sox10). Oligonucleotides with site B and site C/C‘ from the promoter of the *Mpz* (myelin protein zero) gene served as positive control for Sox10 monomer and dimer binding.

Bioinformatic analysis of the OLEa sequence revealed the presence of 14 potential Sox binding sites, labelled I through XIV (Fig. 6E and Fig. 7). In electrophoretic mobility shift assays (EMSA) six sites were found to exhibit strong affinity for Sox10. These were sites I, II, IV, IX, X and XIV (Fig. 6F-P). Site V had a weaker affinity. Sites I and II were closely spaced and allowed binding of a Sox10 dimer. So did sites IX and X, whereas sites IV, V and XIV interacted with a Sox10 monomer (Fig. 6F, H, I, M, P). Each of the sites was mutated in such a way that Sox10 binding was no longer possible (Fig. 8A, C-R) and mutations for the high-affinity sites were introduced into the context of OLEa. Luciferase reporter gene assays in transiently transfected Neuro2A cells showed that mutation of the dimer site IX/X had the largest impact on Sox10 responsiveness among the single site mutations and reduced activation rates from 59-fold to 15-fold (Fig. 8B). The remaining activation rates were even further reduced when mutation of site IX/X was combined with additional mutations of the other sites such as site I/II and site IV. These in vitro studies therefore confirm that Sox10 binds and acts through multiple sites in OLEa.

**Fig 9:**
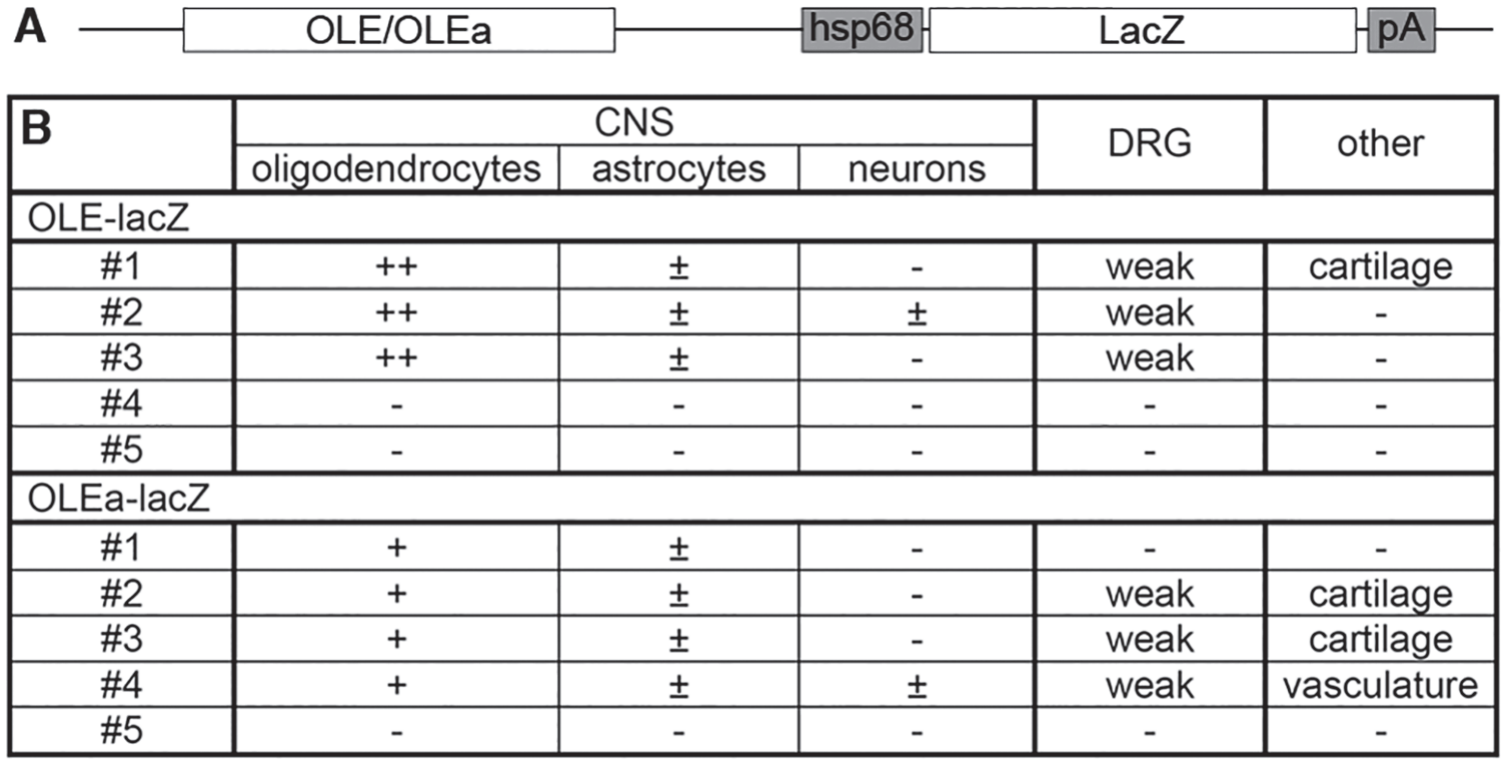
*OLE-lacZ* and *OLEa-lacZ* transgenes are predominantly expressed in oligodendrocytes. **(A)** The transgenic constructs consisting of OLE or OLEa, the minimal Hsp68 promoter (hsp68), the lacZ marker gene (lacZ) and a SV40 polyA signal (pA) are schematically shown. (**B**) Five founders were obtained after pronucleus injection of *OLE-lacZ* and *OLEa-lacZ* that inherited the transgene to their progeny and were expanded to lines. Three of them (OLE#4, OLE#5 and OLEa#5) did not show transgene expression. All other lines exhibited strong and selective expression in oligodendroglial cells with more than 80% of β-galactosidase positive cells in the CNS corresponding to cells of this lineage (+ and ++). In comparison, less than 20% of β-galactosidase positive cells in the CNS were identified as astrocytes (±) in several lines. Only OLE#2 and OLEa#4 exhibited occasional β-galactosidase labelling of neurons (±). A higher fraction of the oligodendroglial population expressed the transgene in lines OLE#1–3 (++) than in OLEa#1–4 (+). Outside the CNS, weak additional transgene expression was observed for several lines within the DRG, in cartilage or in vasculature as indicated.

**Fig 10:**
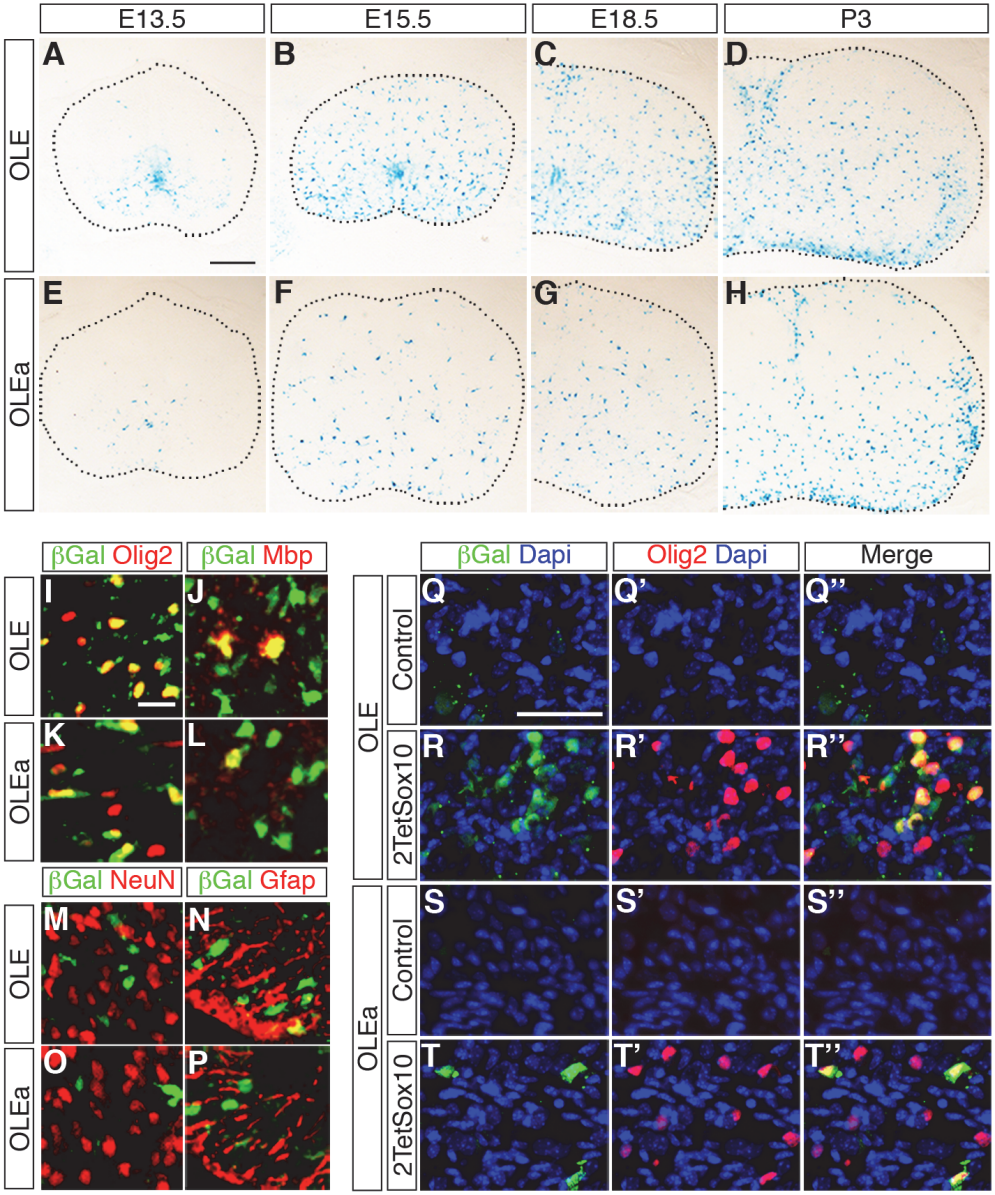
OLE is a Sox10-dependent oligodendroglial enhancer in vivo. **(A-H)** The expression pattern of the lacZ reporter was followed during embryonic and early postnatal development in the spinal cord of *OLE-lacZ* (A-D) and *OLEa-lacZ* (E-H) transgenic animals by X-gal staining of transverse thoracic sections at E13.5, E15.5, E18.5 and P3. Size bar in A, valid for A-H: 200 μm. **(I-P)** Co-IHC was performed on spinal cord tissue of perinatal *OLE-lacZ* (I, J, M, N) and *OLEa-lacZ* (K, L, O, P) transgenic animals using antibodies directed against ß-galactosidase (in green) in combination with antibodies directed against Olig2 (I, K), Mbp (J, L), NeuN (M, O) and GFAP (N, P) (all in red). Pictures were taken from the ventral mantle zone for M, O and from the ventral marginal zone for all other panels. Size bar in I, valid for I-P: 20 μm. **(Q-T”)** Additionally, co-IHC was performed on DRG of mice that carried the *OLE-lacZ* (Q-R”) or the *OLEa-lacZ* (S-T”) transgene on a wildtype (Control) or 2TetSox10 background using antibodies directed against ß-galactosidase (green) in combination with antibodies directed against Olig2 (red). Nuclei were counterstained with Dapi (blue). Shown are single fluorescences for ß-galactosidase (Q-T) and Olig2 (Q’-T’) and the merge (Q”-T”) for each IHC. Size bar in Q, valid for Q-T”: 50 μm.

To analyse whether the identified ECR is active as an oligodendroglial enhancer in vivo, we used *lacZ* reporter gene constructs containing the 2.7kb OLE or its subfragment OLEa in front of a *hsp68* minimal promoter and the reporter gene cassette to generate transgenic mice (Fig. 9A). Five stably transmitting founders were obtained for the *OLE-lacZ* and the *OLEa-lacZ* reporter each (Fig. 9B). Despite some variability among the established lines (Fig. 9B), all exhibited staining in the spinal cord that was compatible with predominant expression in cells of the oligodendrocyte lineage (Fig. 10A-D for *OLE-lacZ* and Fig. 10E-H for *OLEa-lacZ*). Outside the CNS, there was weak transgene expression in the DRG, sometimes accompanied by faint staining in cartilage or vasculature (Fig. 9B).

IHC at E18.5 confirmed the predominantly oligodendroglial expression of the transgene as the majority of β-galactosidase expressing cells were positive for Olig2 and Sox10 at perinatal times (Fig. 10I, K). In contrast, only a small fraction of β-galactosidase expressing cells colabelled with NeuN as a neuronal marker (≤ 5%) or glutamine synthetase and GFAP as astrocytic markers (≤ 20%) (Fig. 10M-P). The substantial overlap between β-galactosidase and Mbp furthermore argues that reporter gene expression is not restricted to OPC but also found in differentiating oligodendrocytes (Fig. 10J, L). Nevertheless, there were some differences between *OLE-lacZ* and *OLEa-lacZ* lines (Fig. 10A-H). The *OLE-lacZ* reporter was on average more widely expressed throughout the oligodendroglial population than the *OLEa-lacZ* reporter. In addition to this better coverage, only OLE, but not OLEa was strongly active in the pMN domain (compare Fig. 10A, B to Fig. 10E, F). This confirms that OLEa contains the key regulatory elements for oligodendroglial activity, but may need to be modified by additional elements present in the larger OLE to faithfully recapitulate the complete developmental expression pattern of Olig2.

We also placed the *OLE-lacZ* and *OLEa-lacZ* transgenes on a background in which 2TetSox10 was expressed under *Sox10*::*Cre*-induced tTA control and investigated reporter gene activation in the DRG (Fig. 10Q-T”). Both transgenic constructs were strongly activated in a subpopulation of cells within the DRG of 2TetSox10 mice (Fig. 10R-R”, T-T”). In agreement with efficacy of transgene expression in the CNS, induction rates varied between the two transgenes and *OLEa-lacZ* transgenic animals reproducibly showed a lower amount of lacZ-expressing cells in their DRG than *OLE-lacZ* transgenic animals. Importantly, lacZ expression was restricted to a subset of the Olig2-expressing cells in the DRG (Fig. 10R, T). This supports the notion that Olig2 expression in DRG of Sox10 overexpressing mice involves the identified *Olig2* enhancer.

**Fig 11:**
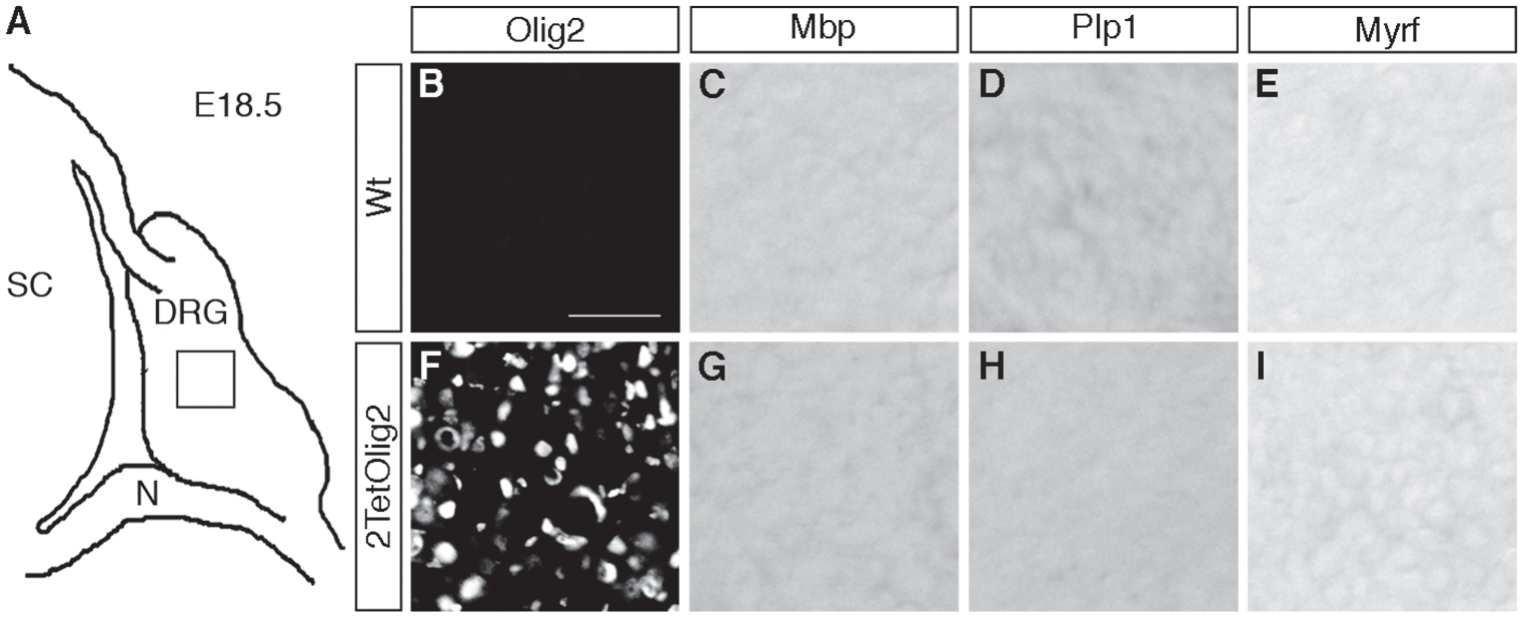
Olig2 overexpression does not generate oligodendrocyte-like cells in DRG. **(A)** Spinal cord (SC), DRG and peripheral nerves (N) are schematically shown at E18.5. **(B-I)** IHC and ISH were carried out on transverse sections (thoracic level) of wildtype (Wt) and 2TetOlig2 embryos at E18.5 with antibodies directed against Olig2 (B, F) and riboprobes against *Mbp* (C, G), *Plp1* (D, H) and *Myrf* (E, I) and. Pictures were taken from the boxed DRG area. Size bars: 50 μm in B (valid for B-I).

Finally we asked whether the presence of Olig2 in DRG glia is sufficient to induce oligodendrocyte-like cells. For that purpose we exchanged the *TetSox10* transgene by an analogously constructed *TetOlig2* transgene [27] and probed the DRG of 2TetOlig2 mice at E18.5 for the expression of the myelin genes *Mbp* and *Plp1* and *Myrf* as a marker for differentiating oligodendrocytes. Unlike 2TetSox10 mice, 2TetOlig2 mice were indistinguishable from the wildtype in that oligodendrocyte markers were not expressed (compare Fig. 11C-E, G-I to Fig. 2C-E, G-I). Therefore Olig2 cannot convert satellite glia into oligodendrocyte-like cells.

## Discussion

Using a genetic strategy that allows transgene expression in all cells that normally express Sox10, we have shown in this study that overexpression of Sox10 in DRG satellite glia is sufficient to directly convert these cells into cells that strongly resemble differentiating oligodendrocytes. Evidence that the reprogrammed cells are oligodendrocyte-like is manifold. These cells express the typical markers and regulatory network components of myelinating oligodendrocytes, including Olig2, its relative Olig1, Nkx2.2 and Myrf. Additionally we find expression of myelin genes such as Mbp and Plp1, and when cultured with DRG neurons, some of these cells acquire the typical morphology of myelinating oligodendrocytes. The presence of Plp1 furthermore shows that the cells are not Schwann cells, which express Mpz instead. Similarly, characteristic regulatory network components of myelinating Schwann cells such as Oct6 and Krox20 were missing from these cells.

Despite their clear oligodendrocyte character these cells were not of CNS origin as evidenced by the fact that CNS-specific overexpression of Sox10 failed to give rise to these cells. Their early appearance in DRG at E12.5 also argues against a CNS origin as it is difficult to imagine that the newly generated OPC could have migrated from the pMN domain all the way through the spinal cord parenchyma into the DRG during this extremely short time window. We also failed to detect substantial levels of Sox9, Pdgfra and NG2 in reprogrammed cells at any time of their development arguing that these cells did not go through a classic OPC stage.

The PNS origin of these oligodendrocyte-like cells was also supported by their appearance after neural crest-wide overexpression of Sox10. Among neural crest-specific cellular sources for these oligodendrocyte-like cells within the PNS boundary cap cells and immature Schwann cells could as much be ruled out as DRG neurons. Instead, Fabp7-positive resident glia within the DRG were identified as the cells in which Olig2 induction occurred. Fabp7 is to date the only reliable marker for satellite glia during embryonic development [16]. We therefore conclude that the oligodendrocyte-like cells arise from satellite glia. However, we are aware that this assignment is based on a single marker and should be revisited once additional markers for embryonic satellite glia become available.

Satellite glia represent a poorly characterized cell population. They are closely apposed to neuronal somata and appear to supply them with nutrients, neurotrophins and other essential molecules. Their intense communication with neurons and strong coupling by gap junctions has led to the assumption that they may be the PNS counterpart to CNS astrocytes [28]. Satellite glia furthermore appear to represent a persistent precursor cell population. They are slowly dividing in the adult and respond to noxious stimuli and inflammation by enhanced proliferation [29]. When taken from their normal environment and placed in culture they have been reported to display plasticity and give rise to various types of PNS and CNS glial cell types [30].

Our finding that satellite glia are prone to reprogramming may thus at least in part be attributable to their precursor cell characteristics and plasticity. To us, the frequency with which satellite glia are converted into oligodendrocyte-like cells upon Sox10 overexpression is particularly noteworthy. With standard in vitro conversion rates (for review, see ref. 2), we would have had little chance to observe this process in vivo. One reason for this phenomenon may actually be found in the microenvironment of satellite glia, including their close proximity to neurons which may supply instructive signals for oligodendrocyte development. However, it is probably also important that satellite glia already express some amount of endogenous Sox10. Considering that Sox10 may function as a pre-patterning factor [31,32], its presence may help to keep those chromatin regions in a poised state that need to be activated during the direct conversion of satellite glia into oligodendrocyte-like cells. It is this activity as pre-patterning factor that makes Sox10 especially suitable for reprogramming strategies. A valuable further property of Sox10 may be its capacity to induce many of the factors that it needs to cooperate with during oligodendroglial cell fate decisions and differentiation processes such as Nkx2.2 and Myrf [8,25].

Equally noteworthy is the fact that reprogramming is achieved by a change of dose rather than introduction of a novel factor. Sox10 amounts are tightly regulated and its functions are concentration-dependent during normal development in mouse and human [16,33,34,35].

One of the results of the increased Sox10 levels in satellite glia is the additional activation of Olig2 as a second essential factor for oligodendrogenesis and oligodendrocyte differentiation. This activation furthermore appears to be direct and mediated by an ECR in the distal upstream region of the *Olig2* gene which is not only active in oligodendroglial cells, but also responds to the presence of Sox10 and is bound by this factor in vitro as well as in vivo. The multiplicity of Sox10 binding sites and the complicated structure of a core and adjacent accessory elements makes this ECR an ideal element for a Sox10 dosage-dependent enhancer that normally comes under Sox10 control in oligodendrocytes when amounts of this transcription factor increase with the onset of differentiation [9], or artificially in satellite glia when Sox10 levels increase by overexpression. This ability of high levels of Sox10 to induce and maintain Olig2 expression is likely a central element in the conversion process as it establishes a key circuit in the corresponding regulatory network. However, our results also indicate that Olig2 induction is not sufficient to convert satellite glia into oligodendrocyte-like cells. This argues that additional Olig2-independent processes are set in motion by high Sox10 levels in satellite glia. The previously reported induction of Myrf expression may be one of them [8].

It is intriguing to assume that other Sox10 expressing neural crest-derived cells with precursor cell characteristics may similarly be convertible into oligodendrocyte-like cells. These include melanocyte stem cells and enteric glia [36]. Especially the latter are similar to DRG satellite glia in their close apposition and functional interaction with neurons, as well as in their maintenance of precursor cell characteristics and plasticity that allows enteric glia to respond to injury with increased proliferation and production of enteric neurons [37]. The presence of melanocyte stem cells and enteric glia in the adult and their relatively easy accessibility may make them amenable to isolation and Sox10-dependent conversion as a realistic source of oligodendrocytes for future applications like cell replacement strategies.

## Materials and Methods

### Plasmids, cell culture, ChIP, transfection, luciferase assays and EMSA

All Plasmids were generated by standard cloning procedures. Expression plasmids for 9myc-tagged Sox10 were based on pCMV5 and pBI-EGFP. Reporter plasmids for transgenic animals were generated by cloning the respective ECR fragments upstream of an *Hsp68* minimal promoter followed by a *lacZ* cassette [38]. For luciferase assays the respective ECR fragments were cloned upstream of a β-globin minimal promoter followed by a luciferase cassette [22], Sox binding sites were mutated using the QuikChange XL site-directed mutagenesis kit (Stratagene). Mouse Neuro2a neuroblastoma cells and rat primary oligodendroglia were kept in culture as described [39]. Transient transfections of Neuro2a cells, luciferase assays and EMSA followed standard procedures [22]. ChIP was performed as reported [31] with the following modifications: Chromatin was prepared from primary oligodendrocytes kept under differentiating conditions for six days and from brain tissue of three week old mice. Fixation was with 1% formaldehyde in PBS. For precipitation of sheared chromatin, anti-Sox10 antiserum and corresponding preimmune serum were used in combination with protein G magnetic beads (Cell Signaling Technology). A list of primers for cloning and detection of genomic fragments in PCR experiments, including their sequence and position is available upon request.

### Generation, husbandry and analysis of transgenic animals, myelinating co-cultures

All animal experiments were carried out with permission and in compliance with animal policies of the local authorities and governmental agencies. Mice transgenic for *TetSox10*, *OLE-lacZ* or *OLEa-lacZ* were obtained by microinjecting the respective linearized DNA into male pronuclei of fertilized oocytes according to standard techniques. Mice transgenic for TetOlig2 have been described [27].

Expression of *TetSox10* was achieved by combining one or two copies of the transgene with the *Sox10* ^*rtTA*^ allele and administration of doxycycline [17]. Alternatively, *TetSox10* expression was induced by a combination of the *Rosa26* ^*stopflox-tTA*^ allele [14] and any of the following Cre alleles: *Sox10*::*Cre* [15], *Wnt1*::*Cre* [40], *Krox20*::*Cre* [41], *Brn4*::*Cre* [20] or *Dhh*::*Cre* [42]. If not otherwise stated, analysed animals contained two copies of the *TetSox10*, and one copy of the *Rosa26* ^*stopflox-tTA*^ and the Cre allele each. Expression of the *TetOlig2* transgene was similarly achieved by combining two copies with one copy of the *Rosa26* ^*stopflox-tTA*^ and the *Sox10*::*Cre* allele.

After genotyping, material from staged embryos was processed for X-Gal staining [9], ISH with probes specific for *Mbp*, *Plp1* and *Myrf*, or IHC using primary antibodies against Sox10 (guinea pig antiserum in 1:1000 dilution) [43], Sox9 (guinea pig antiserum in 1:500 dilution) [44], Glast (guinea pig antiserum in 1:500 dilution, Millipore), Olig1 (rabbit antiserum in 1:10000 dilution, Millipore), Olig2 (rabbit antiserum in 1:1000 dilution, Millipore), Oct6 (rabbit antiserum in 1:2000 dilution) [31], Fabp7 (rabbit antiserum in 1:300 dilution, Millipore), Krox20 (rabbit antiserum in 1:200 dilution, Covance), Mbp (rabbit antiserum in 1:200 dilution, NeoMarkers), β-galactosidase (rabbit antiserum in 1:500 dilution, ICN; goat antiserum in 1:500 dilution, Biotrend), myc-tag (goat antiserum in 1:200 dilution, Abcam), Nkx2.2 (mouse monoclonal in 1:5000 dilution, Developmental Studies Hybridoma Bank, University of Iowa), NeuN (mouse monoclonal in 1:500 dilution, Millipore), Gfap (mouse monoclonal in 1:100 dilution, Millipore), GlnS (mouse monoclonal in 1:1000 dilution, BD Transduction Laboratories), Hb9 (mouse monoclonal in 1:50 dilution, Developmental Studies Hybridoma Bank), Islet1 (mouse monoclonal in 1:1000 dilution, Developmental Studies Hybridoma Bank), and GFP (rat monoclonal in 1:2000 dilution, Nacalai Tesque). Olig1 and Nkx2.2 immunoreactivity were detected with the TSA Plus Cyanine 3 system (PerkinElmer). Source and working concentration of fluorophore-labelled secondary antibodies were as described [10,21,39,45]. Nuclei were counterstained with Dapi.

Coculture experiments were performed as described [46] with the difference that dissociated DRG cells from wildtype or 2TetSox10 mice were added instead of OPC to cultured rat DRG neurons. To this aim, DRG were dissected from E14.5 mouse embroys and dissociated with papain (4 U/ml), DnaseI (40 μg/ml) and L-cysteine (240 μg/ml) at 37°C for 60 min. The dissociated cells were added at a density of 150,000 per well in a 12-well plate and incubated under myelinating conditions for 4 weeks. After fixation, cells were stained with antibodies directed against Mbp (rat monoclonal in 1:750 dilution, Serotec) and Nf165 (mouse monoclonal in 1:3000 dilution, Developmental Studies Hybridoma Bank).

For western blots, brain extracts were prepared as described [47], and proteins were detected with antibodies against Sox10 and Gapdh (Santa Cruz Biotechnology).

### Ethics statement

Mice experiments were in accord with animal welfare laws and approved by the responsible local committees and government bodies (Regierung von Mittelfranken and Behörde für Gesundheit und Verbraucherschutz Hamburg).
